# Supplementary material for: Organic Pollutants Associated with Plastic Debris in Marine Environment: A Systematic Review of Analytical Methods, Occurrence, and Characteristics
Source: Int J Environ Res Public Health. 2023 Mar 10;20(6):4892. doi: 10.3390/ijerph20064892 (PMC10048819; doi:10.3390/ijerph20064892)
Supplement: Supplementary file 1 [file ijerph-20-04892-s001.zip › ijerph-2199222-supplementary.pdf]

Organic Pollutants Associated with Plastic Debris in Marine Environment: A Systematic Review of Analytical Methods, Occurrence, and Characteristics

Hongrui Zhao, Ileana Federigi, Marco Verani and Annalaura Carducci *

Laboratory of Hygiene and Environmental Virology, Department of Biology, University of Pisa, Via S. Zeno 35/39, 56127 Pisa, Italy; hongrui.zhao@phd.unipi.it (H.Z.); ileana.federigi@unipi.it (I.F.); marco.verani@unipi.it (M.V.)

* Correspondence: annalaura.carducci@unipi.it; Tel.: +39-0502213644

Table of contents

Table S1. Main reasons of exclusion of eligible studies 3

Table S2. Description of the reviewed papers (the papers are listed in chronological order, to view the improvements throughout the time) 5

Table S3. Contamination control methods reported in the reviewed papers 36

Table S4. Scoring of the reviewed papers (papers are here listed in descending order of the cumulative score)..39

Table S1. Main reasons of exclusion of eligible studies

Number	Author, Year	Title	Reason of exclusion
1	Bakir A et al., 2012	Competitive sorption of persistent organic pollutants onto microplastics in the marine environment	Lab-scale experiment
2	Barletta et al., 2019	Distribution, sources and consequences of nutrients, persistent organic pollutants, metals and microplastics in South American estuaries	Review
3	Campanale et al., 2020	A Relevant Screening of Organic Contaminants Present on Freshwater and Pre-Production Microplastics	Freshwater (Samples were sourced from freshwater)
4	Dasgupta, et al., 2020	Deep seafloor plastics as the source and sink of organic pollutants in the northern South China Sea	Macroplastics (Samples were primarily composed of macroplastics (> 5mm in size))
5	Dhavamani et al., 2022	Phthalate esters and plastic debris abundance in the Red Sea and Sharm Obhur and their ecological risk level	Irrelevant to MPs-sorbed chemicals research topic (The primary objective was Concentration of PAEs in surface seawater and MPs abundance)
6	Fauvelle et al., 2021	Organic additive release from plastic to seawater is lower under deep-sea conditions	Lab-scale experiment
7	Hairai et al., 2011	Organic micropollutants in marine plastics debris from the open ocean and remote and urban beaches	Macroplastics (Samples were primarily composed of macroplastics (> 5mm in size))
8	Jiang et al., 2023	Plastic debris as a mobile source of additive chemicals in marine environments: In-situ evidence	Irrelevant to MPs-sorbed chemicals research topic (The primary objective was marine invertebrates)
9	Kwon et al., 2017	Microplastics as a vector of hydrophobic contaminants: Importance of hydrophobic additives	Commentary
10	Mendoza et al., 2015	Characterisation of microplastics and toxic chemicals extracted from microplastic samples from the North Pacific Gyre	Macroplastics (Samples were primarily composed of macroplastics (> 5mm in size))
11	Paluselli et al., 2018	Phthalate Release from Plastic Fragments and Degradation in Seawater	Lab-scale experiment

12	Paluselli et al., 2020	Horizontal and vertical distribution of phthalates acid ester (PAEs) in seawater and sediment of East China Sea and Korean South Sea: Traces of plastic debris?	Irrelevant to MPs-sorbed chemicals research topic (Chemicals were extracted from seawater and sediment)
13	Ren et al., 2022	Biphasic Magnetic Levitation to Detect Organic Pollutants on Microplastics	Lab-scale experiment
14	Rios et al., 2007	Persistent organic pollutants carried by synthetic polymers in the ocean environment	Macroplastics (Samples were primarily composed of macroplastics (> 5mm in size))
15	Rios et al., 2010	Quantitation of persistent organic pollutants adsorbed on plastic debris from the Northern Pacific Gyre's ''eastern garbage patch''	Macroplastics (Samples were primarily composed of macroplastics (> 5mm in size))
16	Rochman et al., 2013	Polystyrene Plastic: A Source and Sink for Polycyclic Aromatic Hydrocarbons in the Marine Environment	Lab-scale experiment
17	Santana-Viera et al., 2021	Organic pollutants adsorbed on microplastics: Analytical methodologies and occurrence in oceans	Review
18	Tanaka et al., 2020	Occurrence and concentrations of chemical additives in plastic fragments on a beach on the island of Kauai, Hawaii	Macroplastics (Samples were primarily composed of macroplastics (> 5mm in size))
19	Wu et al., 2016	Sorption of pharmaceuticals and personal care products to polyethylene debris	Lab-scale experiment
20	Zarfl et al., 2010	Are marine plastic particles transport vectors for organic pollutants to the Arctic?	Modeling study

Table S2. Description of the reviewed papers (the papers are listed in chronological order, to view the improvements throughout the time)

Study area	Sampling strategy, sample collection, storage, extraction	MPs abundance/ amount of samples collected or analyzed	Plastic debris size	Polymers	Shape	Most common colours/weathering condition	Mesh size /Filter pore size	Digestion (duration)	Polymer detection method	Chemical detection method	Chemicals sorbed by MPs	Concentration of chemicals	Ref.
Japan; 30 locations across five continents, high-tide line sandy beach; IPW	Selective sampling; pellets were collected visually by soap-rinsed fingers or tweezers. Pellets were wrapped in aluminum foil or paper, put into paper envelops; pellets were sorted immediately after arrival	30 samples of pellets; Around 100 pellets were collected from each beach.	<5 mm	PE, PP, and other polymers; Yellowing PE pellets were subjected to the chemical analysis	Resin pellets (cylinder or disk)	Yellowing pellets (yellowness of 40 or more)	N/R	N/R	NIR, pellets were all analyzed	GC–MS/MS (for PCBs and DDE), GC–ECD (for DDT, DDD and 4 HCH isomers)	PCBs, OCPs (DDTs, and HCHs)	(Median concentration, ng/g-pellet) Σ13PCBs:~300-~600 in the US; ~50-~400 in Japan and western Europe; <50 in south east Asia, Australia, and southern African countries DDTs: ~100-~300 in the US and Vietnam. <20 in other locations.	Ogata et al., 2009

												HCHs: 0.15-37.1	
Portugal; shore line, beach; Compared with IPW	Volume-reduced sampling; Sediment depth: top 2 cm; samples were sieved (2mm metal mesh) in situ and stored in a paper bag; density separation for plastic debris (to determine the size of the micro debris), NaCl, 140 g/L	33 samples analysed	<5 mm	PE，PP and PS	Resin pellets	Aged, black, white and color	2mm metal mesh; 0.45 μm glass microfiber filter	N/R	Micro-FTIR, only representative (the most common plastics) of the debris found in the samples, not all the samples.	GC-MS (only pellets were analyzed for HOCs concentration)	PAHs, PCBs and OCPs (DDTs)	(Individual congener concentration, ng/g) PAHs: 0.2-319.2 PCBs: 0.02-15.56 DDTs: 0.16-4.05	Frias et al., 2010
Greece; High tide line and berm or the upper part,	Selective sampling; yellowing pellets were selected by naked	5 pools (each pool consists of 10 pieces of pellets) were analyzed	N/R	PE, PP, and other polymers.Yellowing PE pellets were subjected	Resin pellets	Yellowing pellets	N/R	N/R	NIR spectrometer，pellets were sorted all	GC–MS/MS	PCBs, OCPs (DDTs, HCHs), and PAHs	(Median concentration, ng/g-pellet) Σ13PCBs：0.13-290 DDTs：1.1-	Karapanagioti et al., 2011

sandy beach	eye by comparison with the reference pellets whose yellowness had been determined by a handy colorimeter; Stored in aluminum foil at 4 °C	for each location		to the chemical analysis								42 HCHs：1.05-3.5 PAHs：100-500	
Japan; Islands in the Pacific, Atlantic, and Indian Oceans and the Caribbean Sea, sandy beach (IPW)	Selective sampling; collected visually by soap-rinsed fingers or tweezers; samples were wrapped in aluminum foil, put into paper envelopes; samples were analyzed	Around 100 pellets were collected from each beach	<5 mm	PE, PP, and other polymers.Yellowing PE pellets were subjected to the chemical analysis	Resin pellets	Yellowing pellets (yellowness of 40 or more)	N/R	N/R	NIR, pellets were all sorted	GC–MS/MS (for PCBs and DDE), GC–ECD (DDT, DDD and 4 HCH isomers)	PCBs, OCPs (DDTs, and HCHs)	(Median concentration, ng/g-pellet) Σ13PCBs: 0.1-9.9 HCHs: 0.2–1.7 Tentative background levels of PCBs < 10, DDTs < 4, and HCHs < 2	Heskett et al., 2012

	immediately upon arrival at the laboratory												
South Africa; beach (IPW)	Volume-reduced sampling; samples were sieved through a 2 mm-mesh sieve and stored in glass vials or jars in a dark cupboard at room temperature	South of Saldanha Bay: 100-700; Blue Water Bay :1000-1800; Woody Cape:1500-10,000 (pellets per linear metre of beach)	N/R	PE (82%), PP (11%), and other polymers (7%);	Resin pellets	Yellowed	2 mm-mesh sieve	N/R	FIR	GC-MS (Yellowing PE pellets were subjected to the chemical analysis)	PCBs, OCPs (DDTs, and HCHs)	(Median concentration, ng/g-pellet) Σ13PCBs (year 1989): 113 (year 2008): 61 HCHs (year 1989)： 112; (year 2008)： 5 DDTs (year 1989)： 1281： (year 2008)： 31	Ryan et al., 2012
United States, San Diego beach	Selective sampling; samples were collected visually by stainless steel tweezers and stored in	2453 individual plastic debris was collected; 1778 pieces were prepared for chemical analysis	68% <5mm	PS	Fragments, PS foam, pellets, and rubber	White, yellowed	Plastic sifter/sieve with <2 mm mesh size	N/R	N/R, because the study did not mention polymer testing, it is questio	GC-MS	PAHs, PCBs, OCPs (DDTs) and chlordanes	(Total concentration, ng/g) PAHs:30-1900 PCBs: ND-47 DDTs: ND-76 chlordanes: 1.8-60	Van et al., 2012

	baked amber glass vials; plastic debris was quantified and separated by color, size and type and stored in a - 20 °C freezer until chemical analysis								nable whether the foam samples they collected were polystyrene.				
Germany; East Frisian Islands, beach sediment	Bulk sampling; Depth: 2 cm surface sediment; metal spoon; samples were placed in PET drinking water bottles (The	38 particles per 10 g sediments and 496 particles per 10 g sediments	10 - 350 µg weight	PE, PP, PS, polyamide 6, chlorinated PE and chlorosulfonated PE	Fibres, fragments	Red, blue,white, and transparent	0.45 µm nitrocellulose filter;	N/R	Py-GC/MS	Py-GC/MS	Organic plastic additives, and PAEs, benzaldehyde (#1, #3) and 2,4-di-tert-butylphenol (#1, #2, #5)	N/R	Fries et al., 2013

	risk of background contamination by phthalates from the PET drinking water sampling bottles is estimated to be low because these OPAs were mainly incorporated into PVC); density separation, NaCl, 1.2 g cm^{-3}												
Portugal; coast, sandy beach (IPW)	Selective sampling; collected visually by soap-rinsed fingers or tweezers; Other informatio	9-pellet sample, 5 pools of pellets (each pool consisting of five randomly selected pellets)	N/R	PE, PP, and other polymers	Resin pellets	Yellowing pellets	N/R	N/R	NIR	GC-MS	PCBs, OCPs (DDTs, HCHs), PAHs, and hopanes	(Median concentration, ng/g-pellet) Σ13PCBs:10.5-307 PAHs: 50-24,000 DDTs：0.0-49	Mizukawa et al., 2013

	n is the same as Ogata et al., 2009;	from each location.										HCHs：0.0-0.86 Hopanes：8250-70,800	
Greece coastal sites; beach sediment	Selective sampling; collected visually by tweezers; samples were then stored in aluminium foil and preserved at 4°C for their transportation to the laboratory; The samples were finally kept at -20 °C until their analysis	Plastic pellets (n = 5)	4-5 mm	N/R	Resin pellets	Erosion yellow	N/R	N/R	N/R, some of the samples were from batches with previously identified polymers, but no proportions were provided	LC-QqLIT-MS/MS-ESI(-)	PFASs	(Total concentration, ng/kg) PFASs: 10-180 PFPeA: 24 - 98	Llorca et al., 2014

Australia and New Zealand; sandy beach (IPW)	Selective sampling; Other information is the same as Ogata et al., 2009	5 pools of pellets (5 pellets for each pool)	<5 mm	PE, PP, and other polymers.Yellowing PE pellets were subjected to the chemical analysis	Resin pellets	Yellowing PE pellets	N/R	N/R	NIR	GC-MS	PCBs, OCPs (DDTs, HCHs)	(Median concentration, ng/g-pellet) Σ13PCBs：0.25-157 in New Zealand ND-294 in Australia DDTs:0.52-421.82 HCHS:ND-28.94	Yeo et al., 2015
China; sandy beach; Compared with IPW	Selective sampling; identified visually by forceps; samples were stored in aluminum foil at 4 °C	220 pellets in total. 22 pellets for chemical analysis.	<5 mm	N/R	Resin pellets	N/R	N/R	N/R	N/R	GC/MS and GC–ECD	PAHs, PCBs and OCPs (HCHs, DDTs, Chlordane, Endosulfan, Heptachlor, Aldrin, Dieldrin, Endrin)	(Total concentration, ng/g) Σ20PCBs: 21.5–323.2 PAHs: 136.3–2384.2 DDTs: 1.15-126.95 Median concentration of ΣOCPs：9.06	Zhang et al., 2015
Vietnam; beach (IPW)	Selective sampling; collected by soap-rinsed fingers; Other	Around 80–100 pellets in total	<5 mm	N/R	Resin pellets	N/R	N/R	N/R	N/R	GC–ECD	PCBs, OCPs (DDTs, HCHs)	(Median concentration, ng/g-pellet) DDTs: 12.3-558 Σ13PCBs:	Le et al., 2016

	information is the same as Ogata et al., 2009											4.0-24.0 HCHs：0.44-1.44	
Brazil; sandy beach	Selective sampling; collected by tweezer; samples were placed in aluminum envelopes	Three randomly selected pools (replicates) of 1 g for each resin were analyzed	1-5mm	PE and PP	Resin pellets	Ranging in tone from near-white to dark red	1 mm sieve	N/R	N/R. Density separation of two polymers (water + ethanol solution). metioned about RAMAN spectroscopy	GC-MS	PAHs	(Total concentration, ng/g) ΣPAHs of PE pellets：737 - 39,763 ΣPAHs of PP pellets: 871 - 9252	Fisner et al., 2017
South Korea; Korean coast sandy beach and Asia-Pacific coastal region	Selective sampling; collected by tweezer and stainless steel knives; collected samples were immediat	36 microplastic samples in South Korea. 46 marine debris in other areas.	2-3 mm	Expanded polystyrene (EPS)	Debris	N/R	N/R	N/R	N/R	HPLC-MS	BFRs (HBCD)	(Concentration, μg/g) HBCD: mean:1070 ± 450, median: 912; range: 751–2700 in South Korea range: 0.98-14,500 in	Jang et al., 2017

	ely wrapped with aluminum foil, and stored at -20 °C until analysis											Asia-Pacific coastal region	
South Korea; beach	N/R (no detailed sampling strategy); Each individual product was also photographed to illustrate its use. All samples were covered with aluminium foil and stored at −20 °C before chemical analysis	29 plastic debris	N/R	PE, PP, PET, PC, and acrylic/styrene	Debris	N/R	N/R	N/R	FTIR	LC-MS/MS	Plastic additives - UV stabilizers and antioxidants, UV 320, UV 326, UV 327, UV 328, Irganox 1076, Irganox 1010, BHT, and 2,4-DTBP	(Total concentration, µg/g) Antioxidant: 0.21 - 1620 (mean ± sd: 143± 287; median: 93) UV stabilizers: 0.003 - 82 (mean ± sd: 8.3 ± 19, median: 0.53)	Rani et al., 2017

Greece; sandy beach, sediment	Volume-reduced sampling; The samples were collected by hand using forceps and stored in 500 mL glass containers and kept in the dark and refrigerated until analyzed; two monitoring campaigns	Analoukas beach (1195 plastic pellets/m^2 and 1197.5 fragments/m^2)； Petres beach (4.4 plastic pellets/m^2 and 2.5 fragments/m^2)	4–15 mm（most abundant）	N/R	pellets and fragments	white, old white, off-white, orange, brown, colored	2 mm mesh sieve	N/R	N/R	GC-ITMS	PAHs	(Individual congener concentration, ng/g) PAH range: ND -1592 First campaign:1.3 -329 in pellets; 1.3 - 636.5 in plastic fragments Second campaign:1 to 1049 in pellets; 0.5-1592 in fragments Average Σ16PAHs: First campaign: 405.7 in pellets; 536.6 in plastic fragments Second campaign: 1782 in pellets; 1300 in plastic fragments	Karkanorachaki et al., 2018

China; surface seawater	Volume-reduced sampling; all retained debris (as collected in the cod-end) were transferred to a 500 mL glass bottle; Samples were stored at 2-4 ℃ until analysis	3 to 162 particles per 100 m^3	0.33-5 mm	PE and PS	Line, foam, fragment, pellet and others	N/R	0.333 mm mesh manta trawl; stainless steel sieves of 10, 40, and 50 meshes	Validated the effectiveness of hydrogen peroxide pretreatment	ATR-FTIR	GC-MS	PAHs	(Total concentration, ng/g) Σ16PAHs: 3400-120,000	Mai, L. et al. 2018
China; coastal beach	Bulk sampling; collected by shovel; density separation ($Li_2O_{13}W_4{}^{-}{}_{24}$ density=1.6 g/mL) solution); the samples were placed in self-sealing	28 sand and sediment samples, 41 microplastic samples	＜5mm	PE, PP and PS	Fragment, flake, foam, pellet	N/R	N/R	N/R	ATR-FTIR	GC-MS and GC-TQMS	PAEs	(Total concentration, ng/g) Σ9 PAEs: 0–80.4 Total average concentrations： PAEs: 1.53	Zhang et al., 2018

	bags and taken back to the laboratory, where they were kept in a clean, light-proof place and air-dried at room temperature.												
Spain; beach	Volume-reduced sampling; collected by metal spoon, samples were placed in a 1 mm mesh bag, identified and separated with forceps. The samples were dried at	133 samples of pooled pellets	Large micro-debris (1–5 mm), and meso-debris or mesoplastic (5–25 mm)	PE (95%) and PP (5%)	Pellets and fragments	N/R	1 mm mesh bag	N/R	FTIR	GC-TQMS	PAHs, PCBs, OCPs, UV-filters, BFRs (PBDE, BDEs), and chlorpyrifos (a kind of OPs)	(Total concentration, ng/g, data of mesoplastics are not included) ΣPAHs: 52.1–17,023.6 ΣPCBs: 0.9–2285.8 ΣOCPs: 0.4–13,488.7 ΣUV-filters: 0 - 3,740.3 ΣBDEs：0–180.58	Camacho et al., 2019

	room temperature (about 25°C)											Chlorpyrifos: median 3.1; range 0.5 to 48.4	
North Pacific Subtropical Gyre; surface seawater	Volume-reduced sampling; A manta trawl with a mouth opening of 15 cm × 90 cm; samples were placed inside zip-lock bags or wrapped in aluminum and were then frozen at −2 °C for transport to the laboratory	N/R	Small (0.5 - 1.5 mm), medium (1.5-5 mm), and large (5 mm-15 mm) plastics	PE	Hard fragments	N/R	Manta trawl, mesh size 500 µm	N/R	µ-FTIR	UPLC-TQMS-(ESI-)	EDCs (BPAs, BPS, NPs)	(Average concentration, µg/kg) BPAs: 475 ± 882 Bisphenol S ： 7.3 ± 25.9 Octylphenol: 2.5 ± 8.7 Nonylphenol: 3.7 ± 7.7	Chen et al., 2019

Brazil; beach (IPW)	Selective sampling; collected by metal tweezers; samples were placed into foil envelopes, identified with parchment-paper labels and kept frozen at -20 °C until analyses	14 replicate stations (60 pellets for each station)	1-5 mm	N/R	Resin pellets	N/R	N/R	N/R	N/R	GC-MS	PAHs and PCBs	(Total concentration, ng/g) PAHs:1,454-6,020 PCBs: 0.8-104.6	Gorman et al., 2019
Spain; Mediterranean beach	Selective sampling; samples were obtained manually; All samples were refrigerated in amber glass bottles and were frozen	21.3 ± 18.1 items/m^2 in La Llana beach and 128 items/m^2 in Calblanque beach	MPs (<0.5 cm), mesoplastics (0.5–2.5 cm) and macroplastics (>2.5 cm)	PS, PE PA, and PP	Fragment, pellet	N/R	N/R	N/R	ATR-FTIR, most common polymers were selected	GC-MS	PAH, PCPs, current use pesticides, organochlorinated compounds (including PCBs and OCPs), plastic additives	(Mean concentration, ng/g plastic, dw) PAHs: 17.75-27.40 OCPs: 1.66-12.64 PCBs: b.q.l.-0.30 Triazines: 0.35-1.67 OPPs: 2.12-15.00 Other Pestisides:	León et al., 2019

	(−20 °C) until analysis											1.36-25.34 PCPs: 57.10-161.04 Plastic additives: 51.39-205.26	
China; Hong kong sandy beach	Volume-reduced sampling; density separation in-situ using ambient seawater with addition of NaCl until saturated; the samples were stored on ice during transportation to the laboratory where they were stored at −20 °C	Samples from ten sandy beaches	0.25–5 mm	PE, PP, PET, PS and PVC	Pellet and microbead	N/R	250 μm sieve	N/R	FTIR	GC-MS	PAHs, PCBs, OCPs	(Total concentration, ng/g, dw) ΣPAHs: 70.8 - 1,509, mean: 435 ΣPCBs: 13 - 1,083 ΣOCPs: 48.1-770 DDT and its metabolites (ΣDDX): 1.960-626 ΣHCHs: 5.02-63.5	Lo et al., 2019

	until analysis												
Gulf of Guinea in the tropical Atlantic Ocean beach	Volume-reduced sampling; a stainless-steel spoon was used to scoop the samples; NaCl Density separation; samples were wrapped in aluminium foil and stored in clean Ziploc bags; the retained materials in the sieve were	3424 particles per m^2	1–5 mm	PET(41%), PS(28%), PP(21%),PA(5%), PUR(3%), EVA(1%), PE(0.5%)	Pellets and fibres	Yellow, white, green, black, green, brown, purple, etc	Stainless-steel sieves with mesh sizes 5 mm, 3 mm and 1 mm,	N/R	FTIR	GC-Q-MS	PAEs	(Total concentration, mg/kg dw) Σ6PAEs: BDL - 164.09	Benson et al., 2020

	rinsed with distilled water and air dried at room temperature of 28 °C.												
Taiwan; southwestern coast, surface seawater	Volume-reduced sampling; samples were collected by fishing boat trawl; water samples were stored in pre-cleaned glass jars and temporarily kept in buckets. All samples were shipped to the laboratory	Range: 0.10-0.86 items/$m^{3,}$ average: 0.36 ± 0.21 items/m^3	0.33 -5 mm	PE, PP, PS ，PA and PVC	Fragments: (66.1 ± 10.6%), films (24.0 ± 8.6%), lines (5.7 ± 4.4%), foams (1.7 ± 1.9%), and spheres (2.4 ± 2.5%)	White, black, colored, transparent	Manta trawl, 0.330 mm mesh; 5 mm stainless steel screen; 0.6 µm glass fiber filter.	35% H_2O_2 digestion	ATR-FTIR	GC-MS	PAHs	(Total concentration, ng/g, dw) PAHs: 104 - 3,595 average: 818 ± 874	Chen et al., 2020

	for further processing on the day of sampling; saturated NaCl density separation												
China; riverine outlets, surface water	Volume-reduced sampling; samples were collected by a manta net; the samples were stored in brown glass bottles and sealed with glass lids; the bottled samples were cooled at 2–4 °C in a walking refrigerat	133 samples	0.3-5 mm	PE (51.1%) PP (37.5%), PA, PVC	Fragments,pellets and lines	N/R	Manta trawl, 330 μm mesh size	N/R	ATR-FTIR	GC-MS	PAHs, BFRSs (PBDEs) and PCBs	(Mean concentration, ng/g, dw) Σ16PAH: 2010; range: 25–40,100 Σ8PBDE: 412; range: 0.84–14,800 Σ14PCB: 67.7; range: 1.86–456	Mai et al., 2020

	or until analysis												
Chile; coastal beach	Selective sampling; samples were visually collected; No storage information reported	370 samples	4.0 ± 0.6 mm	HDPE (99%)，PP	Resin pellets	New, white (32%), aged, yellowing (68%)	N/R	N/R	ATR-FTIR	GC-MS	BFRs (PBDEs), PCBs and OCPs	(Total concentration, ng/g-pellet) Σ10PBDEs: 10 - 133 Σ7PCBs: 3-60 DDTs: 0.1-7.4	Pozo et al., 2020
China; river mouth, coastal beach	Bulk sampling; sediment samples were collected by stainless-steel shovel; density separation process (distilled water); the samples were kept	N/R	mainly 1.19–5 mm	N/R	Fragments (60%), foam (27%), and pellets (13%).	N/R	N/R	N/R	N/R	GC-MS	PAHs and OCPs	(Total concentration, ng/g) ΣPAH: 11.2 - 7710 ΣOCP: 2.2 - 1970	Shi et al., 2020

	at −20 °C covered with aluminum foil												
Iran; Persian Gulf beach (IPW)	Selective sampling; Other information is the same as Ogata et al., 2009	At each location, 50 to 100 yellowed pellets were collected	< 5 mm	PE, PP and others	Resin pellets	Yellowing PE pellets	N/R	N/R	NIR	GC-MS	PCBs, PAHs and hopanes	(Median concentration, ng/g-pellet) Σ27PAH: 273-15,786 Σ13PCBs: 54–624 Hopanes: 8,048–59,778	Alidoust et al., 2021
Italy; surface seawater	Volume-reduced sampling; floating samples were collected by a manta trawl; Samples were rinsed from the outside to the end of the net, placed in	Highest abundance : 1.88 ± 1.78 items/m³ 3.42 ± 2.28 items/m³ average: 2.65 items/m³	Mainly < 5 mm	N/R	Spherical, filament, fragment, sheet, other shape	White (37%), transparent (44%), blue, red, black, green, other colour	300 µm manta trawl.	N/R	N/R	GC-MS	PCBs, PAHs, OCPs, and OPs	(Total concentration, ng/g) ΣPCB: 1.86 - 65.67 ΣOPs: 4.72-147.61 ΣOCP1s: 1.03-101.26 ΣOCp2s: 5.05-77.79 ΣPAHs: 18.98-408.06	Capriotti et al., 2021

	glass containers and immediately stored at 4 ℃												
China; river drain outlets, surface water	Volume-reduced sampling; all the plastic-like debris captured by the trawl were transferred to a 1-L brown bottle; after each sampling event, the samples were transported to laboratories and stored at 4 ℃ prior to analysis;	Range: 0.003 - 2.09 items/m^3 average: 0.106 items/m^3	0.33-5 mm	PP (43.1%), PE (39.1%) and PS (10.6%)	Fragment (21.2%), fiber (53.1%), film (13.1%), foam (11.4%) and pellet (1.27%)	N/R	0.33mm manta trawl; stainless steel sieve, 50 meshes	30% H_2O_2 digestion	ATR-FTIR	HPLC-MS/MS	PFASs	(Total concentration, ng/g, dw) PFASs：3.11 - 9.07 × 10^3; average: 616	Cheng et al., 2021

	saturated NaCl density separation; five monitoring campaigns												
China; coastal wetlands, surface water and wetland surface wet sediment	Bulk sampling; stainless steel tweezers and plastic buckets were used for transferring samples; samples were wrapped with aluminum foil in an oven under 65 °C for drying for the preparation to isolate the	0.1 - 19.5 items/L in water and 0–1366.7 items/kg in sediment	0.5-5 mm	PP (44.7%), PS (28.5%), PE (20.3%), PES, PU and PVC	Fragment	Mainly black, blue, transparent, gray and white	20 µm nylon filter	H_2O_2 digestion	µ-FTIR	Py/TD-GC-MS	PAEs and BFR (PBDEs)	(Total concentration, ng/g) Σ(7)PAEs: 26.8-4241.8 µg/g Σ(9)PBDEs: no detectable - 250.1 µg/g	Deng et al., 2021

	microplastics from sediment. Saturated NaCl density separation and H_2O_2 digestion of organic matter												
United States, beach	Selective sampling; samples were collected by solvent-rinsed metal forceps, and stored in 25 mL amber glass vials at 4 ◦C until analysis	5 subsamples, each with 20 nurdles	1–5 mm	Mainly PE (81.9%) and PP (18.1%)	Resin pellets	Transparent, white, yellow, black, red, green, and blue	N/R	N/R	FTIR	GC-MS	PAHs and PCBs	(Total concentration, ng/g) Σ16 PAHs: 1.6 - 14,699.8 ng/g Σ7 PCBs: 0 - 642.4 ng/g	Jiang et al., 2021

China; western Hong Kong beach	Volume-reduced sampling; samples were collected in a pre-cleaned stainless steel bucket; all the MPs were picked under a stereomicroscope and freeze-dried, subsequently stored at − 20 ◦C until further analysis	MP samples from 11 sandy beaches	< 5mm	PP (40.7 ± 15.1%), PE (38.6 ± 13.9%), PS (13.6 ± 11.0%) and PET (1.53 ± 3.12%).	Particles	N/R	125 µm sieve	N/R	µ-FTIR	LC-MS/MS	EDCs (BPA, BPB, BPF and BPS)	(Mean concentration, ng/g) BPA: 82.4–989 ng/g	Lo et al., 2021
Beaches across 27 countries (IPW)	Selective sampling, sampels were selected by stainless steel tweezers or clean	Five pools of pellets (each comprising five randomly selected pellets)	< 5mm	PE, PP, and others.	Resin pellets	Yellowing PE pellets	N/R	N/R	NIR	GC–IT/MS and GC-ECD	BFR (PBDEs) and PCBs	(Total concentration, ng/g-pellet) ΣPBDEs: 0.08-46, median: 2.0 Σ13PCBs: 0.15–2230, median: 51	Ohgaki et al. 2021

	hands and wrapped in aluminum; Upon arrival, the pellets were kept frozen at – 30°C until analysis												
Spain; beach	Volume-reduced sampling; samples was collected and sieved through a 1mm mesh; samples were carefully manipulated in the laboratory using tweezers to remove other materials; no information about	Twenty samples were analysed	1mm-5mm	N/R	Pellets and fragments	N/R	1 mm mesh sieve	N/R	N/R	UPLC-MS/MS	UVFs and UVSs	(Total concentration, ng/g) ΣUVFs: 1 - 4031	Santana-Viera et al., 2021

	sample storage												
Taiwan; beach	Volume-reduced sampling; samples were pick up to glassware by a tweezer; Other information is the same as Ogata et al., 2009	N/R	< 5mm	N/R	Resin pellets	N/R	Stainless steel sieves (1 mm)	N/R	N/R	HRGC-HRMS	POPs, including: polychlorinated dibenzo-p-dioxins and -furans, PBDEs, PCBs, PBBs, and their congeners.	(Total concentration, surface of MPs, Cs; overall concentrations within MPs, Ct) PCDD/Fs (Cs): 1.92-14.59 pg/g ; (Ct): 95.0-1110.6 pg/g PBDD/Fs (Cs): N.D.–1388.0 pg/g ; (Ct): 3092–29,343 pg/g PBDEs (Cs): 0.228–319.99 ng/g; (Ct): 7.0–3888.6 ng/g PCBs (Cs):	Wang et al., 2021

												0.04–0.57 ng/g; (Ct): 0.27–5.7 ng/g PBBs (Cs): N.D.–41.91 pg/g; (Ct): N.D.–202.2 pg/g	
Spain; seawater of Mar Menor Lagoon	Bulk sampling; 2L of seawater (top 5 cm) were collected and stored in amber glass bottles; Large debris were visually separated by sieving; The bottles were transported under	0.13 - 9,303 ng/mL in summer 4,918 and 8,744 ng/mL in winter	< 20 mm	PS, PE, PI, PBD, PP, PA, and PVC	Debris	N/R	Stainless sieve (20 μm mesh); 0.7 μm glass microfiber filters	N/R	LC-SEC-HRMS	LC-HRMS	Plasticizers (phthalates group); stabilizers (antioxidants, e.g., distearyl 3,30-thiodipropionate, 2,5-di-tert-butylhydroquinone), and UV filters as benzotriazoles. Flame retardants (group of phosphates). Pharmaceuticals, pesticides, food additives,	N/R	Vega-Herrera et al., 2021

	cool conditions to the lab and stored at 4 °C										flavors and natural products. 74 of the 135 chemicals were additives.		
Iran; Persian Gulf coastline, Urban runoff water, river estuary	Bulk sampling; samples were taken and stored in pre-cleaned glass vessels (with a volume of 5 L) and covered with aluminum foil; saturated NaI (density =1.6 g/cm^{-3}) density separation; filter residuals were	1.86 items/L (mean)	500-1000 µm	N/R	Fiber (54%), fragment, film, round shape	Black, red, blue, white/transparent and green/yellow	5 mm mesh sieve, 300-5000 µm mesh screen	30% H_2O_2 digestion	N/R	GC-MS	PAEs	(Concentration, µg/L) PAEs: 53.57 µg/L (Average); 25.32-137.54 µg/L (mean range)	Hajiouni et al., 2022

	stored in a glass storage dish at -18 °C												
Nigeria; beach	Volume-reduced sampling; samples were collected by stainless-steel spoon; sample was wrapped in aluminium foil and stored in clean Ziploc bags. Each sample was air dried; NaCl solution density separation	Range: 0.01 to 0.77 items/m^2 average: 0.25 ± 0.20 items/m^2	1–5 mm	PE, PS, PP, PET, PUR, PA , PEVA	Fragments, PS foam, pellets, and rubber	N/R	5 mm mesh stainless steel sieves;1 mm sieve	N/R	ATR-FTIR	GC-Q-MS	PAHs, PCBs and OCPs	(Total concentration, mg/kg) ΣPAHs: 0.00-0.32 ΣPCBs: 0.00–0.53 ΣOCPs: 0.04–2.02	Fred-Ahmadu et al., 2022

N/R = not recorded within the study; ND = not detectable; dw = dry weight. BFRs = brominated flame retardants; BPA = bisphenol A; BPB = bisphenol B; BPF = bisphenol F; BPS = bisphenol S; BDEs = bromodiphenyl ethers; DDT = dichlorodiphenyltrichloroethane; EVA = ethyl vinyl acetate; EDCs = endocrine-disrupting chemicals; ATR-FTIR = attenuated total reflection Fourier transform infrared spectroscopy; FIR = far-infrared spectroscopy; GC-ECD = GC with electron capture detector; GC-IT/MS = gas chromatography-ion trap mass spectrometry; GC-MS = chromatography-mass spectrometry; GC-MS-MS = gas chromatography-tandem mass spectrometry; GC-Q-MS = gas chromatography with quadrupole mass; GC-TQMS = gas chromatograph-triple quadrupole mass spectrometer; H_2O_2 = hydrogen peroxide; HBCD = hexabromocyclododecane; HRGC-HRMS = high-resolution gas chromatograph/high-resolution mass spectrometry; HPLC-MS = high-performance liquid chromatography-mass spectrometry; HPLC-MS/MS = high-performance liquid chromatography with tandem mass spectrometry; IPW = International Pellet Watch; LC-HRMS = liquid chromatography coupled to high resolution mass spectrometry; LC-MS/MS = liquid chromatography with tandem mass spectrometry; LC-QqLIT-MS/MS-ESI (-) = liquid chromatography coupled to quadrupole-linear ion trap tandem mass spectrometer equipped with a Turbo Ion Spray source operating in negative electrospray ionisation mode; NIR = near-infrared spectroscopy; OPAs = organic plastic additives; OCPs = organochlorine pesticides; OPs = organophosphorus; OPEs = organophosphorus esters; OPFRs = organophosphorus flame retardants; PAHs = polycyclic aromatic hydrocarbons; PAEs = phthalates; PBDEs = polybrominated diphenyl ethers; PBBs = polybrominated biphenyls; PBDFs = polybrominated dibenzofurans; PBDDs = polybrominated dibenzo-p-dioxins; PE = polyethylene; PEVA = Poly(ethylene-co-vinyl acetate); PET = polyethylene terephthalate; PI = polyisoprene; PBD = polybutadiene; PA = polyamide; PCBs = polychlorinated biphenyls; PCPs = personal care products; PP = polypropylene; PS = polystyrene; PUR = polyurethane; PVC = polyvinylchloride; Py-GC-MS = pyrolysis-gas chromatography-mass spectrometry; Py/TD-GC-MS = pyrolyzer/thermal desorption gas chromatography-mass spectrometry; UPLC-MS/MS = ultra-performance liquid chromatography-tandem mass spectrometry; UPLC-TQMS-(ESI-) = ultra-performance liquid chromatography-triple quadrupole mass spectrometry equipped with an electrospray ionization source operating in negative mode; UVFs = UV filters; UVSs = UV stabilizers; PFASs = perfluoroalkyl substances; PFPeA = perfluoropentanoic.

Table S3. Contamination control methods reported in the reviewed papers

Publication country	Quality assurance and quality control	Reference
Japan	Along with each set of samples (5 pellet pools), a procedural blank (for chemical analysis) was analyzed.	Ogata et al., 2009
Portugal	At all times contact with plastic materials was avoided to prevent contamination.	Frias et al., 2010
Japan	Along with each set of samples (5 pellet pools), a procedural blank (for chemical analysis) was analyzed.	Karapanagioti et al., 2011
Japan	Along with each set of samples (5 pellet pools), a procedural blank (for chemical analysis) was analyzed.	Heskett et al., 2012
South Africa	Along with each set of samples (5 pellet pools), a procedural blank (for chemical analysis) was analyzed.	Ryan et al., 2012
United States	Along with each sequence of samples (10 samples), a procedural blank (for chemical analysis) was analyzed.	Van et al., 2012
Germany	Procedural blank (for laboratory air cross-contamination prevention); all samples and materials were washed (dishwasher or hand wash) and covered with aluminium foil before they were first used prior to testing. The materials and vessels used were covered with aluminium foil after each single step. Lab coats were worn throughout the analysis. The analysis site was cleaned prior to opening and analysing the petri dishes containing the samples.	Fries et al., 2013
Japan	Along with each set of samples (5 pellet pools), a procedural blank (for chemical analysis) using only solvent was analyzed.	Mizukawa et al., 2013
Spain	Blank samples (cross-contamination prevention) during transport, preservation and analysis were processed in parallel with the collected samples; handlers wear PFAS-free latex gloves in all cases.	Llorca et al., 2014
Japan	Along with each set of samples (5 pellet pools), a procedural blank (for chemical analysis) using only solvent was analyzed.	Yeo et al., 2015
China	Blank samples were analyzed (chemical analysis); all devices were thoroughly rinsed with dichloromethane before and after analysis and sample preparation was carried out in a ultra clean laboratory.	Zhang et al., 2015
Malaysia	Along with each set of samples (5 pellet pools), a procedural blank (for chemical analysis) was analyzed.	Le et al., 2016
Brazil	N/R, (No specific description of cross-contamination control and no negative control)	Fisner et al., 2017
Korea	Procedural blanks (chemical analysis); The inner part of each EPS sample was used for analysis to avoid contamination, pre-cleaned sampling tools were used.	Jang et al., 2017
Korea	Procedural blanks (chemical analysis) were analyzed; samples were covered with aluminium foil to avoid contamination.	Rani et al., 2017
Greece	All equipment was cleaned with deionised water and acetone before use; samples were stored in clean containers.	Karkanorachaki et al., 2018

China	Three procedural blanks (chemical analysis) were prepared for each sample site; Sampling tools were thoroughly rinsed with distilled water prior to each sampling event to minimise cross contamination.	Mai, L. et al. 2018
China	Three procedural blanks (chemical analysis) were analyzed along with the samples.	Zhang et al., 2018
Spain	Reagent blank (chemical analysis).	Camacho et al., 2019
China	Procedural blanks, positive control and solvent control (chemical analysis).	Chen et al., 2019
Brazil	Extraction blanks (chemical analysis) and a surrogate-recovery experiment.	Gorman et al., 2019
Spain	Procedural blanks (chemical analysis)) were analyzed along with the samples.	León et al., 2019
China	Procedural blanks (chemical analysis) were analyzed for every four samples.	Lo et al., 2019
Nigeria	Reagent blanks and procedural blanks (chemical analysis); in order to avoid cross-contamination of laboratory items, strict attention was paid to the experimental procedures.	Benson et al., 2020
China	Procedural blanks (chemical analysis); the sampling tools were rinsed with seawater.	Chen et al., 2020
China	Five procedural blanks and forty field blanks (cross-contamination prevention).	Mai et al., 2020
Czech Republic	Procedural blanks (chemical analysis).	Pozo et al., 2020
China	One procedural blank, duplicate samples, and one standard reference material sample (chemical analysis) were analyzed for every ten field samples.	Shi et al., 2020
Japan	A procedural blank (for chemical analysis) using only solvent was analyzed.	Alidoust et al., 2021
Italy	To prevent contamination, the laboratory procedures were performed according to Baini et al.,2018: sampling tools were carefully rinsed prior to use. In the laboratory procedure, glassware was used and samples were analyzed in a clean airflow cabinet to prevent air contamination; two glass Petri dishes placed on either side of the stereo microscope were used as blank controls.	Capriotti et al., 2021
China	Field blanks, procedural blanks (field cross-contamination prevention and chemical analysis) and matrix-spiked samples (cross-contamination prevention); sampling tools were thoroughly rinsed with distilled water; nitrile gloves and cotton lab coats were worn during the entire procedures of experiments. Containers and tools were rinsed three times with methanol until dry and wrapped in aluminium foil before use.	Cheng et al., 2021
China	50 blanks (lab cross-contamination prevention) were run to evaluate the background contamination. Plastic-free products were used during sampling and analysis process; the entire process of MPs identification was conducted in a clean laboratory. Drawbacks: Plastic buckets were used for surface water sampling; Nylon filters were used for filtering.	Deng et al., 2021

United States	Two empty glass bottles as blank (chemical analysis) for each sampling site were analyzed	Jiang et al., 2021
China	One procedural blank (chemical analysis) was carried out after each batch of 8 samples to check for contamination.	Lo et al., 2021
Japan	Along with each set of samples (5 pellet pools), a procedural blank (for chemical analysis) using only solvent was analyzed.	Ohgaki et al. 2021
Spain	N/R (No specific description of cross-contamination control and no negative control)	Santana-Viera et al., 2021
China	Plastic-free sampling tools and containers were used, and gloves were worn during the sampling process to prevent cross-contamination.	Wang et al., 2021
Spain	For LC analysis, 1.5L of water was used as a blank (chemical analysis); in order to monitor contamination from instrumental analysis, by injecting solvent blanks of toluene stochastically with each injection samples list in the polymeric analysis or plastic additives in toluene, and methanol in methanolic fraction analysis.	Vega-Herrera et al., 2021
Iran	A solvent blank, procedural blank (chemical analysis) were prepared with each batch of samples; only glassware was used in the experiments; all equipment was rinsed with ultrapure water before use; plastic-free lab coats and gloves were used throughout the sampling and analysis; a control glass petri dish containing distilled water was put on a clean cabinet in the laboratory, and blank samples (to prevent cross-contamination in the laboratory) were covered with clean aluminium foil during the analysis step.	Hajiouni et al., 2022
Nigeria	Blank samples (cross-contamination prevention) of saturated NaCl solution were filtered and examined; Procedural blanks (chemical analysis) were carried out along with certified standard; all samples were wrapped in aluminum foil sheets and stored at 4 °C before analysis.	Fred-Ahmadu et al., 2022

Table S4. Scoring of the reviewed papers (papers are listed in descending order of the cumulative score)

Article	Sampling methods	Sample amount	Sample storage	Contamination control	Sample extraction	Polymer identification	Detection of chemicals	Total Score
Cheng et al., 2021	3	3	3	3	3	3	3	21
Fred-Ahmadu et al., 2022	3	3	3	3	3	3	3	21
Chen et al., 2020	3	3	3	2	3	3	3	20
Mai, L. et al. 2018	3	3	3	2	3	3	3	20
Benson et al., 2020	3	3	3	2	3	3	3	20
Deng et al., 2021	3	3	2	2	3	3	3	19
Ryan et al., 2012	3	3	3	1	3	3	3	19
León et al., 2019	3	3	3	1	3	3	3	19
Hajiouni et al., 2022	3	3	3	3	3	0	3	18
Ogata et al., 2009	3	2	3	1	3	3	3	18
Karapanagioti et al., 2011	3	2	3	1	3	3	3	18
Heskett et al., 2012	3	2	3	1	3	3	3	18
Mizukawa et al., 2013	3	2	3	1	3	3	3	18
Llorca et al., 2014	3	2	3	2	3	2	3	18
Yeo et al., 2015	3	2	3	1	3	3	3	18
Zhang et al., 2018	3	2	3	1	3	3	3	18
Camacho et al., 2019	3	2	3	1	3	3	3	18
Lo et al., 2019	3	2	3	1	3	3	3	18
Mai et al., 2020	3	2	3	1	3	3	3	18
Alidoust et al., 2021	3	2	3	1	3	3	3	18
Jiang et al., 2021	3	2	3	1	3	3	3	18
Ohgaki et al. 2021	3	2	3	1	3	3	3	18
Vega-Herrera et al., 2021	3	3	3	2	3	3	1	18
Fries et al., 2013	3	3	2	2	3	3	1	17
Zhang et al., 2015	3	3	3	2	3	0	3	17
Frias et al., 2010	3	1	3	1	3	3	3	17
Lo et al., 2021	3	1	3	1	3	3	3	17

Capriotti et al., 2021	3	3	3	1	3	0	3	16
Van et al., 2012	3	3	3	1	3	0	3	16
Karkanorachaki et al., 2018	3	3	3	1	3	0	3	16
Chen et al., 2019	3	0	3	1	3	3	3	16
Jang et al., 2017	3	2	3	2	3	0	3	16
Pozo et al., 2020	3	2	0	1	3	3	3	15
Le et al., 2016	3	2	3	1	3	0	3	15
Rani et al., 2017	1	2	3	2	1	3	3	15
Gorman et al., 2019	3	2	3	1	3	0	3	15
Fisner et al., 2017	3	2	3	0	3	1	3	15
Shi et al., 2020	3	0	3	1	3	0	3	13
Wang et al., 2021	3	0	3	1	3	0	3	13
Santana-Viera et al., 2021	3	1	0	0	3	0	3	10
**Average Score**	**2.95**	**2.15**	**2.80**	**1.35**	**2.95**	**2.10**	**2.90**	**17.20**
**Median Score**	**3**	**2**	**3**	**1**	**3**	**3**	**3**	**18**

References

1. Alidoust, M.; Yeo, G.B.; Mizukawa, K.; Takada, H. Monitoring of polycyclic aromatic hydrocarbons, hopanes, and polychlorinated biphenyls in the Persian Gulf in plastic resin pellets. *Mar. Pollut. Bull.* **2021**, *165*, 112052, doi: 10.1016/j.marpolbul.2021.112052.
2. Benson, N.U.; Fred-Ahmadu, O.H. Occurrence and distribution of microplastics-sorbed phthalic acid esters (PAEs) in coastal psammitic sediments of tropical Atlantic Ocean, Gulf of Guinea. *Sci. Total Environ.* **2020**, *730*, 139013, doi: 10.1016/j.scitotenv.2020.139013.
3. Camacho, M.; Herrera, A.; Gómez, M.; Acosta-Dacal, A.; Martínez, I.; Henríquez-Hernández, L.A.; Luzardo, O.P. Organic pollutants in marine plastic debris from Canary Islands beaches. *Sci. Total Environ.* **2019**, *662*, 22-31, doi: 10.1016/j.scitotenv.2018.12.422.
4. Capriotti, M.; Cocci, P.; Bracchetti, L.; Cottone, E.; Scandiffio, R.; Caprioli, G.; Sagratini, G.; Mosconi, G.; Bovolin, P.; Palermo, F.A. Microplastics and their associated organic pollutants from the coastal waters of the central Adriatic Sea (Italy): Investigation of adipogenic effects in vitro. *Chemosphere* **2021**, *263*, 128090, doi: 10.1016/j.chemosphere.2020.128090.
5. Chen, C.F.; Ju, Y.R.; Lim, Y.C.; Hsu, N.H.; Lu, K.T.; Hsieh, S.L.; Dong, C.D.; Chen, C.W. Microplastics and their affiliated PAHs in the sea surface connected to the southwest coast of Taiwan. *Chemosphere* **2020**, *254*, 126818, doi: 10.1016/j.chemosphere.2020.126818.
6. Chen, Q.; Allgeier, A.; Yin, D.; Hollert, H. Leaching of endocrine disrupting chemicals from marine microplastics and mesoplastics under common life stress conditions. *Environ. Int.* **2019**, *130*, 104938, doi: 10.1016/j.envint.2019.104938.
7. Cheng, Y.; Mai, L.; Lu, X.; Li, Z.; Guo, Y.; Chen, D.; Wang, F. Occurrence and abundance of poly- and perfluoroalkyl substances (PFASs) on microplastics (MPs) in Pearl River Estuary (PRE) region: Spatial and temporal variations. *Environ. Pollut.* **2021**, *281*, 117025, doi: 10.1016/j.envpol.2021.117025.
8. Deng, H.; Li, R.; Yan, B.; Li, B.; Chen, Q.; Hu, H.; Xu, Y.; Shi, H. PAEs and PBDEs in plastic fragments and wetland sediments in Yangtze estuary. *J. Hazard. Mater.* **2021**, *409*, 124937, doi: 10.1016/j.jhazmat.2020.124937.
9. Fisner, M.; Majer, A.; Taniguchi, S.; Bícego, M.; Turra, A.; Gorman, D. Colour spectrum and resin-type determine the concentration and composition of Polycyclic Aromatic Hydrocarbons (PAHs) in plastic pellets. *Mar. Pollut. Bull.* **2017**, *122*, 323-330, doi: 10.1016/j.marpolbul.2017.06.072.
10. Fred-Ahmadu, O.H.; Tenebe, I.T.; Ayejuyo, O.O.; Benson, N.U. Microplastics and associated organic pollutants in beach sediments from the Gulf of Guinea (SE Atlantic) coastal ecosystems. *Chemosphere* **2022**, *298*, 134193, doi: 10.1016/j.chemosphere.2022.134193.
11. Frias, J.P.; Sobral, P.; Ferreira, A.M. Organic pollutants in microplastics from two beaches of the Portuguese coast. *Mar. Pollut. Bull.* **2010**, *60*, 1988-1992, doi: 10.1016/j.marpolbul.2010.07.030.
12. Fries, E.; Dekiff, J.H.; Willmeyer, J.; Nuelle, M.T.; Ebert, M.; Remy, D. Identification of polymer types and additives in marine microplastic particles using pyrolysis-GC/MS and scanning electron microscopy. *Environ. Sci.: Process. Impacts* **2013**, *15*, 1949-1956, doi: 10.1039/c3em00214d.
13. Gorman, D.; Moreira, F.T.; Turra, A.; Fontenelle, F.R.; Combi, T.; Bícego, M.C.; de Castro Martins, C. Organic contamination of beached plastic pellets in the South Atlantic: Risk assessments can benefit by considering spatial gradients. *Chemosphere* **2019**, *223*, 608-615, doi: 10.1016/j.chemosphere.2019.02.094.
14. Hajiouni, S.; Mohammadi, A.; Ramavandi, B.; Arfaeinia, H.; De-la-Torre, G.E.; Tekle-Röttering, A.; Dobaradaran, S. Occurrence of microplastics and phthalate esters in urban runoff: A focus on the Persian Gulf coastline. *Sci. Total Environ.* **2021**, 150559, doi: 10.1016/j.scitotenv.2021.150559.
15. Heskett, M.; Takada, H.; Yamashita, R.; Yuyama, M.; Ito, M.; Geok, Y.B.; Ogata, Y.; Kwan, C.; Heckhausen, A.; Taylor, H.; et al. Measurement of persistent organic pollutants (POPs) in plastic resin pellets from remote islands: Toward establishment of background concentrations for International Pellet Watch. *Mar. Pollut. Bull.* **2012**, *64*, 445-448, doi: 10.1016/j.marpolbul.2011.11.004.
16. Jang, M.; Shim, W.J.; Han, G.M.; Rani, M.; Song, Y.K.; Hong, S.H. Widespread detection of a brominated flame retardant, hexabromocyclododecane, in expanded polystyrene marine debris and microplastics from South Korea and the Asia-Pacific coastal region. *Environ. Pollut.* **2017**, *231*, 785-794, doi: 10.1016/j.envpol.2017.08.066.
17. Jiang, X.; Lu, K.; Tunnell, J.W.; Liu, Z. The impacts of weathering on concentration and bioaccessibility of organic pollutants associated with plastic pellets (nurdles) in coastal environments. *Mar. Pollut. Bull.* **2021**, *170*, 112592, doi: 10.1016/j.marpolbul.2021.112592.
18. Karapanagioti, H.K.; Endo, S.; Ogata, Y.; Takada, H. Diffuse pollution by persistent organic pollutants as measured in plastic pellets sampled from various beaches in Greece. *Mar. Pollut. Bull.* **2011**, *62*, 312-317, doi: 10.1016/j.marpolbul.2010.10.009.
19. Karkanorachaki, K.; Kiparissis, S.; Kalogerakis, G.C.; Yiantzi, E.; Psillakis, E.; Kalogerakis, N. Plastic pellets, meso- and microplastics on the coastline of Northern Crete: Distribution and organic pollution. *Mar. Pollut. Bull.* **2018**, *133*, 578-589, doi: 10.1016/j.marpolbul.2018.06.011.
20. Le, D.Q.; Takada, H.; Yamashita, R.; Mizukawa, K.; Hosoda, J.; Tuyet, D.A. Temporal and spatial changes in persistent organic pollutants in Vietnamese coastal waters detected from plastic resin pellets. *Mar. Pollut. Bull.* **2016**, *109*, 320-324, doi: 10.1016/j.marpolbul.2016.05.063.

21. León, V.M.; García-Agüera, I.; Moltó, V.; Fernández-González, V.; Llorca-Pérez, L.; Andrade, J.M.; Muniategui-Lorenzo, S.; Campillo, J.A. PAHs, pesticides, personal care products and plastic additives in plastic debris from Spanish Mediterranean beaches. *Sci. Total Environ.* **2019**, *670*, 672-684, doi: doi.org/10.1016/j.scitotenv.2019.03.216.
22. Llorca, M.; Farré, M.; Karapanagioti, H.K.; Barceló, D. Levels and fate of perfluoroalkyl substances in beached plastic pellets and sediments collected from Greece. *Mar. Pollut. Bull.* **2014**, *87*, 286-291, doi: 10.1016/j.marpolbul.2014.07.036.
23. Lo, H.S.; Po, B.H.K.; Li, L.; Wong, A.Y.M.; Kong, R.Y.C.; Li, L.; Tse, W.K.F.; Wong, C.K.C.; Cheung, S.G.; Lai, K.P. Bisphenol A and its analogues in sedimentary microplastics of Hong Kong. *Mar. Pollut. Bull.* **2021**, *164*, 112090, doi: 10.1016/j.marpolbul.2021.112090.
24. Lo, H.S.; Wong, C.Y.; Tam, N.F.; Cheung, S.G. Spatial distribution and source identification of hydrophobic organic compounds (HOCs) on sedimentary microplastic in Hong Kong. *Chemosphere* **2019**, *219*, 418-426, doi: 10.1016/j.chemosphere.2018.12.032.
25. Mai, L.; Bao, L.J.; Shi, L.; Liu, L.Y.; Zeng, E.Y. Polycyclic aromatic hydrocarbons affiliated with microplastics in surface waters of Bohai and Huanghai Seas, China. *Environ. Pollut.* **2018**, *241*, 834-840, doi: 10.1016/j.envpol.2018.06.012.
26. Mai, L.; He, H.; Bao, L.J.; Liu, L.Y.; Zeng, E.Y. Plastics Are an Insignificant Carrier of Riverine Organic Pollutants to the Coastal Oceans. *Environ. Sci. Technol.* **2020**, *54*, 15852-15860, doi: 10.1021/acs.est.0c05446.
27. Mizukawa, K.; Takada, H.; Ito, M.; Geok, Y.B.; Hosoda, J.; Yamashita, R.; Saha, M.; Suzuki, S.; Miguez, C.; Frias, J.; et al. Monitoring of a wide range of organic micropollutants on the Portuguese coast using plastic resin pellets. *Mar. Pollut. Bull.* **2013**, *70*, 296-302, doi: 10.1016/j.marpolbul.2013.02.008.
28. Ogata, Y.; Takada, H.; Mizukawa, K.; Hirai, H.; Iwasa, S.; Endo, S.; Mato, Y.; Saha, M.; Okuda, K.; Nakashima, A.; et al. International Pellet Watch: Global monitoring of persistent organic pollutants (POPs) in coastal Waters. 1. Initial phase data on PCBs, DDTs, and HCHs. *Mar. Pollut. Bull.* **2009**, *58*, 1437-1446, doi: 10.1016/j.marpolbul.2009.06.014.
29. Ohgaki, T.; Takada, H.; Yoshida, R.; Mizukawa, K.; Yeo, B.G.; Alidoust, M.; Hirai, N.; Yamashita, R.; Tokumaru, T.; Watanabe, I.; et al. International pellet watch: Global monitoring of polybrominated diphenyl ethers (PBDEs) in plastic resin pellets. *Environ. Monit. Contam. Res.* **2021**, *1*, 75-90, doi: 10.5985/emcr.20210002.
30. Pozo, K.; Urbina, W.; Gómez, V.; Torres, M.; Nuñez, D.; Přibylová, P.; Audy, O.; Clarke, B.; Arias, A.; Tombesi, N.; et al. Persistent organic pollutants sorbed in plastic resin pellet - "Nurdles" from coastal areas of Central Chile. *Mar. Pollut. Bull.* **2020**, *151*, 110786, doi: 10.1016/j.marpolbul.2019.110786.
31. Rani, M.; Shim, W.J.; Han, G.M.; Jang, M.; Song, Y.K.; Hong, S.H. Benzotriazole-type ultraviolet stabilizers and antioxidants in plastic marine debris and their new products. *Sci. Total Environ.* **2017**, *579*, 745-754, doi: 10.1016/j.scitotenv.2016.11.033.
32. Ryan, P.G.; Bouwman, H.; Moloney, C.L.; Yuyama, M.; Takada, H. Long-term decreases in persistent organic pollutants in South African coastal waters detected from beached polyethylene pellets. *Mar. Pollut. Bull.* **2012**, *64*, 2756-2760, doi: 10.1016/j.marpolbul.2012.09.013.
33. Santana-Viera, S.; Montesdeoca-Esponda, S.; Sosa-Ferrera, Z.; Santana-Rodríguez, J.J. UV filters and UV stabilisers adsorbed in microplastic debris from beach sand. *Mar. Pollut. Bull.* **2021**, *168*, 112434, doi: 10.1016/j.marpolbul.2021.112434.
34. Shi, J.; Sanganyado, E.; Wang, L.; Li, P.; Li, X.; Liu, W. Organic pollutants in sedimentary microplastics from eastern Guangdong: Spatial distribution and source identification. *Ecotoxicol. Environ. Saf.* **2020**, *193*, 110356, doi: 10.1016/j.ecoenv.2020.110356.
35. Van, A.; Rochman, C.M.; Flores, E.M.; Hill, K.L.; Vargas, E.; Vargas, S.A.; Hoh, E. Persistent organic pollutants in plastic marine debris found on beaches in San Diego, California. *Chemosphere* **2012**, *86*, 258-263, doi: 10.1016/j.chemosphere.2011.09.039.
36. Vega-Herrera, A.; Llorca, M.; Savva, K.; León, V.M.; Abad, E.; Farré, M. Screening and Quantification of Micro(Nano)Plastics and Plastic Additives in the Seawater of Mar Menor Lagoon. *Front. Mar. Sci.* **2021**, *8*, doi: 10.3389/fmars.2021.697424.
37. Wang, L.C.; Lin, J.C.; Dong, C.D.; Chen, C.W.; Liu, T.K. The sorption of persistent organic pollutants in microplastics from the coastal environment. *J. Hazard. Mater.* **2021**, *420*, 126658, doi: 10.1016/j.jhazmat.2021.126658.
38. Yeo, B.G.; Takada, H.; Taylor, H.; Ito, M.; Hosoda, J.; Allinson, M.; Connell, S.; Greaves, L.; McGrath, J. POPs monitoring in Australia and New Zealand using plastic resin pellets, and International Pellet Watch as a tool for education and raising public awareness on plastic debris and POPs. *Mar. Pollut. Bull.* **2015**, *101*, 137-145, doi: 10.1016/j.marpolbul.2015.11.006.
39. Zhang, H.; Zhou, Q.; Xie, Z.; Zhou, Y.; Tu, C.; Fu, C.; Mi, W.; Ebinghaus, R.; Christie, P.; Luo, Y. Occurrences of organophosphorus esters and phthalates in the microplastics from the coastal beaches in north China. *Sci. Total Environ.* **2018**, *616-617*, 1505-1512, doi: 10.1016/j.scitotenv.2017.10.163.
40. Zhang, W.; Ma, X.; Zhang, Z.; Wang, Y.; Wang, J.; Wang, J.; Ma, D. Persistent organic pollutants carried on plastic resin pellets from two beaches in China. *Mar. Pollut. Bull.* **2015**, *99*, 28-34, doi: 10.1016/j.marpolbul.2015.08.002.
